# A Survey of Current Practices and Adherence to Coronavirus Disease Airway Management Guidelines Among Frontline Healthcare Professionals in a Resource-Limited Country

**DOI:** 10.5152/TJAR.2021.21104

**Published:** 2022-10-01

**Authors:** Amber Gulamani, Faisal Shamim, Sehrish Khan, Sohaib Hashmi, Shemila Abbasi

**Affiliations:** 1 Department of Anaesthesiology, The Aga Khan University Hospital, Karachi, Pakistan; 2 Department of Emergency Medicine, The Aga Khan University Hospital, Karachi, Pakistan

**Keywords:** Airway management, COVID-19, healthcare workers, safe airway practices

## Abstract

**Objective:**

The coronavirus disease 2019 (COVID-19) has brought anaesthesiologists, intensive care and emergency physicians to the forefront due to their airway management skills. The aim of survey was to determine current practice trends in COVID-19 airway management among frontline healthcare professionals of Pakistan and their adherence to standard principles proposed by most consensus guidelines.

**Methods:**

An online questionnaire was designed based on consensus guidelines from international societies. We contacted consultants and trainees nationwide working in anaesthesia, intensive care, and emergency departments through emails, phone calls, and social media platforms.

**Results:**

A total of 285 individuals participated in this cross-sectional descriptive study. Intubations were largely performed by anaesthetists followed by emergency physicians. Deteriorating respiratory failure (89%) was the most frequent indication. Availability of trained staff, use of intubation checklist, limited staff presence during intubation, and use of appropriate personal protective equipment were positive findings. One-third reported that their workplace did not have negative pressure rooms for aerosol-generating procedures, and 63.3% responders do not perform airway assessment before intubation. The device of choice for the first attempt at laryngoscopy was Macintosh laryngoscope (51.6%) followed by videolaryngoscopes with disposable blades (24.2%). Availability of rescue devices in case of unanticipated difficult airway is variable; laryngeal mask airway (70.1%), bougie (82.2%), and stylet (68.7%) were present at majority places. Frequency of airway-related adverse events including hypoxemia (69.8%) and failed first attempt intubation (35.2%) was significant.

**Conclusion:**

This survey found satisfactory knowledge, comparable practices, and offers some important insights about airway management in COVID-19 patients by healthcare professionals of Pakistan.

## Main Points

- Tracheal intubation of a coronavirus disease 2019 (COVID 19) patient is a crucial event not only for the staff involved but also for the critically ill patient with little physiological reserves.
- Videolaryngoscope has been proposed as a first-line device for tracheal intubation in COVID-19, but it is not universally available in resource-limited countries.
- Institutions caring for COVID-19 patients should focus on reducing airway management complications by developing their own guidelines and checklists.

## Introduction

World Health Organization (WHO) declared coronavirus disease 2019 (COVID-19) as a pandemic on March 11, 2020, with 118 319 confirmed cases in 114 countries and 4292 reported deaths.^1^ Since then, the severity of the outbreak has exponentially increased and seriously hampered healthcare systems all over the world. Studies from the epicenters of the disease including China, the United States, and Italy found that 5%-22% of these patients developed critical illness characterized primarily by acute respiratory distress syndrome, the majority of whom required invasive mechanical ventilation.^2-4^

Airway management in an acutely ill, decompensating patient is challenging. The incidence of difficult airway, equipment failure, and adverse events such as hypoxemia, circulatory collapse, esophageal intubation, and aspiration is quite high.^5^ Amid the COVID-19 pandemic, airway management has become even more critical as tracheal intubation exposes involved personnel to high viral load and subsequently elevates the risk for viral transmission.^3^ Several organizations have formulated guidelines for safe airway management in these patients to address the gravity of the problem.^6-8^ However, even the finest of healthcare systems are not able to fully cope due to lack of appropriate personal protective equipment (PPE), overburden of healthcare personnel, poor infrastructure, and shortage of medical supplies.^9^

Pandemic posed a unique challenge to low-middle income countries like Pakistan due to fragile healthcare infrastructure, dense population, and limited resources. But the nation has fought back fairly well despite predictions of impending doom.^10^ Reasons for the mysterious flattening of the curve are still under speculation. With third-wave underway, evaluation of practices of the most at-risk population in our healthcare setup that includes anaesthesiologists, intensive care and emergency physicians is important to prepare for the future.

We surveyed healthcare professionals responsible for airway management in COVID-19 patients across Pakistan to assess their practices with regard to standard guidelines^6,8^ and problems faced during intubation of COVID patients at remote locations outside operating rooms.

## Methods

The study was exempted from the Ethics Review Committee (Ref #2020-5251-11453, dated July 27, 20). An online survey was built on Google Forms comprising 34 questions in 3 sections: demographics, airway management practices in COVID-19 patients, and problems/adverse events faced during tracheal intubation. Structured questions were based on consensus guidelines for managing the airway in patients with COVID-19 formulated by Difficult Airway Society, Association of Anaesthetists, the Intensive Care Society, Faculty of Intensive Care Medicine, and the Royal College of Anaesthetists.^8^ Consent to participate was sought at the beginning of the questionnaire. Confidentiality of the participant and data was maintained by anonymous format of the form.

A sample size of 276 was required to estimate the expected rate within 5% margin of error and 95% CI assuming 1000 healthcare workers were directly involved in airway management of COVID-19 patients and 50% of them follow safe practices as per international guidelines. The target duration to complete sample size was 3 months. The link to the Google form was disseminated by investigators through emails, specialty-specific groups on WhatsApp and Twitter. Also, professional colleagues were requested to disseminate the survey in their respective departments.

## Statistical Analysis

Data were exported to Statistical Package for the Social Sciences version 19 (SPSS Inc., Chicago, IL, USA) for analysis. All responses were reported as percentages and complied in charts.

## Results

A total of 285 responses were analyzed. The sample size was achieved in 36 days. A total of 281 (98.6%) respondents consented for use of anonymous information for research purposes and were directed to complete further questions. Demographic profile of the participants is presented in Table 1. 

**Figure 1.**
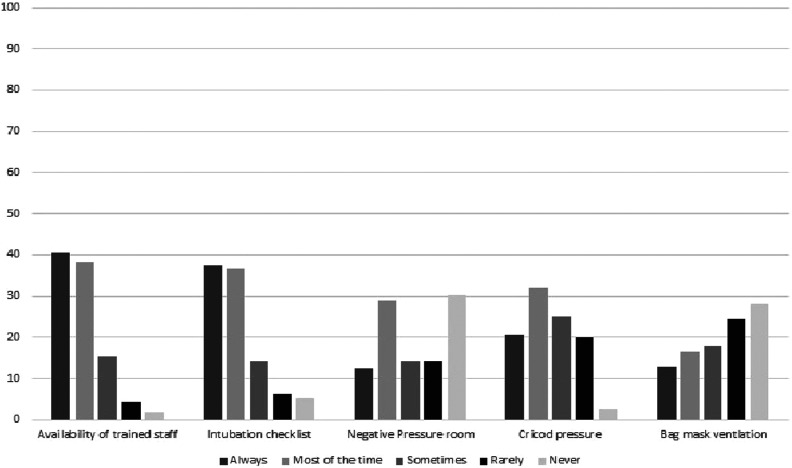
Use of precautionary measures for intubation during the pandemic.

Among healthcare professionals, anaesthesiologists performed most tracheal intubations in institutions across Pakistan for COVID-19 patients and were done by both consultants (54.1%) and trainees (59.4%). A total of 89% respondents had to perform emergency intubation in deteriorating respiratory failure, followed by urgent/planned intubation for impending respiratory failure (61.9%) and increasing oxygen requirement (45.2%). Trained technician/staff help was always available to 40.6% healthcare professionals. In total, 37% participants always used an intubation checklist. Only 12.5% have negative pressure room every time for intubation (Figure 1). Personal protective equipment use by participants is summarized in Table 2. 

The majority of healthcare professionals (63.3%) do not perform airway assessment before intubation and rely only on experience and clinical judgment. Likewise, composite airway assessment scores are not commonly employed in practice. Only 61 (20%) professionals reported use of an airway score, the most common among them is Look, Evaluate the 3-3-2 rule, Mallampati score, Obstruction, and Neck mobility (LEMON) score (65.6%). In case of an anticipated difficult airway, only 21% responders marked cricothyroid membrane for front of neck airway, 26.7% marked it occasionally, and 52.3% have never marked cricothyroid membrane.

**Figure 2.**
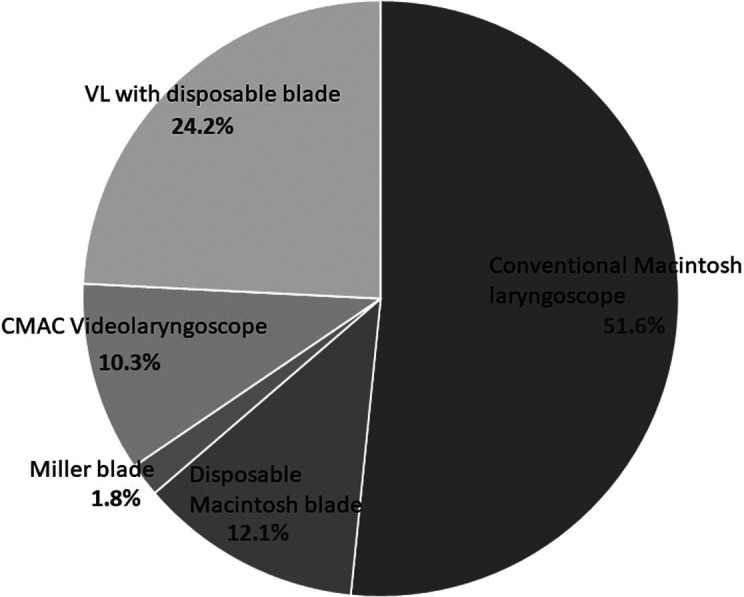
The device of choice for the first attempt at laryngoscopy. Conventional Macintosh laryngoscope (51.6%), videolaryngoscope with disposable blade (24.2%), laryngoscope with disposable Macintosh blade (12.1%), videolaryngoscope (CMAC) (10.3%), and Miller straight blade (1.8%).

The preferred technique of holding face mask varies among the professionals; 34.9% have only used single-handed technique, 42.3% prefer 2 hands C-E technique, and 22.3% have used both hands V-E grip technique. Classic rapid sequence intubation involving an intravenous anesthetic/sedative and rapid-acting muscle relaxant is the most favored technique (67.3%). We received mixed responses for bag-mask ventilation before the first attempt at tracheal intubation (Figure 1). Conventional Macintosh laryngoscope is the most used device for first attempt laryngoscopy (51.6%) (Figure 2). Combination of methods employed for confirmation of endotracheal tube placement includes direct visualization (72.2%), bilateral chest rise (75.8%), carbon dioxide detector (39.1%), and chest auscultation (59.4%). In case of unanticipated difficult airway, equipment commonly available are laryngeal mask airway (70.1%), I-gel (38.1%), bougie (82.2%), ETT stylet (68.7%), other laryngoscope blades, for example, McCoy, Miller (29.2%), and videolaryngoscope (44.5%). Professionals also reported the use of additional protective barriers like intubation shield box (45.9%), clear plastic drape (35.9%), airway management tent (2.8%), and suction-assisted laryngoscopy airway device (4.3%). In total, 51% of professionals reported the presence of only 3 healthcare providers including the intubator in the patient’s room at the time of intubation and the average time spent in patient’s room was less than 20 minutes. Compliance with important guidelines reported by our survey is summarized in Table 3.

**Figure 3.**
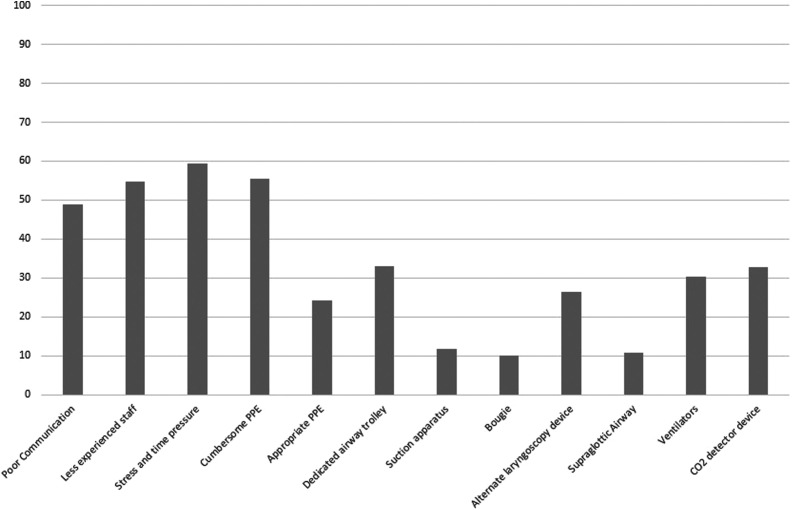
Reported frequencies of problems related to human factors and unavailability of resources.

Problems faced during tracheal intubation of COVID patients related to human factors and equipment are illustrated in Figure 3. The most common respiratory adverse event reported in our survey was significant hypoxemia (69.8%). The frequency of airway-related adverse events was also significant: failed the first attempt at intubation (35.2%), dislodgement of endotracheal tube (ETT) during patient transportation (22.8%), esophageal intubation (19.9%), endobronchial intubation (10.7%), and laryngospasm (11%). Cardiovascular adverse events reported include significant hypotension that required treatment with vasopressors (58%), cardiac arrest (40.2%), and arrhythmias (22.1%).

## Discussion

Tracheal intubation in critically ill COVID-19 patients is technically complex and needs the expertise of the most skilled clinician as endorsed by majority of the guidelines.^6-8^ Same as our survey finding, published literature from around the world confirms that anaesthesiologists are the most common physicians performing emergency tracheal intubations for COVID-19 patients due to their extensive training in airway management.^11-13^ Many anaesthesia departments have created special intubation teams in their institutions and have achieved extraordinary success in ensuring patient and staff safety.^14^

Availability of trained staff, use of intubation checklist, and effective infection control measures like appropriate use of PPE and limited staff presence during airway management were positive findings in our survey. Implementation of these infection control measures by the frontline workers worldwide is reflected in relatively low COVID-19 infection rates in this high-risk group. Among staff enrolled in Intubate COVID-19 study, 3.1% tested positive for COVID-19 and 0.1% were hospitalized for it.^13^ In a study assessing the effect of PPE availability on COVID-19 seroprevalence in frontline staff serving in teaching hospitals of Peshawar-Pakistan, staff who received PPE in time at the start of the pandemic had less chances of contracting the infection (odds ratio = 0.96).^15^

An important concern identified in our survey is the lack of airway assessment practice before intubation. Airway examination is vital especially in COVID patients as it may allow timely preparation for a difficult airway situation and prevent multiple attempts. However, elaborate airway assessment is not always possible in critically ill patients as a result of life-threatening emergency due to hypoxia or inability of the patient to cooperate for assessment because of sedation or unconsciousness.^16^ Short and easy-to-use scoring systems can help the clinicians identify high-risk patients. Mallampati score III or IV, Obstructive Sleep Apnea Syndrome, Reduced Mobility of Cervical Spine, Limited Mouth Opening, Coma, Severe Hypoxia, Non-Anaesthesiologist Intubator (MACOCHA) score though not widely used is validated in critically ill patients and is recommended in COVID-19 patients.^8,17,18^

Meticulous pre-oxygenation with well-fitting mask (preferably 2 handed V-E grip) via closed circle circuit at low flow for 3-5 minutes and refraining from bag-mask ventilation when possible is considered ideal.^8^ On the other hand, our survey found a significant deviation from recommended practice among the participants likely due to intubations done in acutely hypoxic patients outside operating rooms where anesthesia machines are not available. 

Evidence suggests that videolaryngoscope improves the glottic view, reduce the number of failed intubations even in patients with difficult airway, and increases the safe distance between the patient and the intubator.^19,20^ Global data indicate that 80% of intubations in COVID patients were performed with videolaryngoscopes.^20^ However, only a quarter of our cohort preferred videolaryngoscope as their first choice. But, in case of unanticipated difficult airway, videolaryngoscope was available to nearly half of them for the second attempt. This may be due to personal choice of Macintosh blade or unavailability of videolaryngoscope on-site, and it may have to be transferred from some other place like operating room. The use of Macintosh blade as the first choice for laryngoscopy by our cohort also explains the high frequency of failed first attempt and esophageal intubations. This finding is consistent with a Chinese study on emergency intubations in COVID-19 patients, where first-pass success rate with Macintosh blade was 70%.^16^

Most participants confirmed tube placement under direct vision and by bilateral chest rise as waveform capnography is not widely available in remote areas. The same methods were employed by airway managers in a study on COVID intubations performed in an intensive care unit.^16^ Besides, more than half of the participants also used chest auscultation. Unavailability of capnography at remote locations could be the reason to still employ chest auscultation for ETT confirmation. However, chest auscultation is not reliable in patients with respiratory complications of COVID-19 and can be a source of spread of infection and hence not endorsed.^8,16^ Use of portable colorimetric capnometers may be more rational in these situations.

Tracheal intubation is a crucial event not only for the staff involved but also for the critically ill COVID-19 patients with very little physiological reserves. Our survey results regarding adverse respiratory, airway and circulatory events are in accordance with reported literature. In a descriptive study^[Bibr b11-tjar-50-5-346]^ including 202 COVID-19 patients in China, hypoxemia (SpO_2_ <90%) occurred in 73.3% and hypotension (<90/60 mm Hg) was observed in 17.8% patients during and was observed in 22.3% after intubation.^11^ Another study^[Bibr b16-tjar-50-5-346]^ from China reported that 39% patients were hypotensive and required vasopressor support after intubation.^16^ Several participants have witnessed cardiac arrest during intubation in the present survey, though the incidence of peri-intubation cardiac arrest in critically ill patients reported in the literature is 2.7%.^21^ A plausible explanation for this is the high incidence of hypoxemia and circulatory instability in these patients. Also, intubations might have been performed at an advanced stage of disease and patients were more likely to collapse. COVID-19 has taken a toll on the mental health of healthcare professionals. Stress and time pressures are a common concern in our survey. A recent survey evaluating barriers among healthcare professionals in Pakistan in managing COVID-19 patients reported a high frequency of burnout symptoms in healthcare workers.^22^

Several limitations of the study were recognized. First, the survey was distributed through personal contacts and acquaintances. We were not able to formally contact every hospital, as contact information was not readily available on websites and networking among institutions is not quite robust. Secondly, the survey may be filled by individuals working in the same institute and may show repetition of practices. Third, individual intubations were not observed; therefore, the true incidence of adverse events could not be reported. Also, intensivists made very little contribution to the survey, which may reflect the fact that they are not readily involved in airway management. Most ICUs have anaesthesiologist cover to deal with airway-related emergencies. 

This survey offers an important insight into airway management practices for COVID patients in Pakistan. It is a low-middle income country and national guidelines must be developed that should address improving the outcomes of these high-risk patients with limited resources.

## Figures and Tables

**Table 1.** Demographic Profile of the Participants

	Respondents n (%)
**Gender**	
Male	176 (62.6)
Female	105 (37.4)
**Primary clinical specialty**	
Anaesthesiology	184 (65.5)
Intensive care	11 (3.9)
Emergency medicine	79 (28.1)
Internal medicine	6 (2.1)
Pulmonology	1 (0.4)
**Years of clinical experience (years)**	
Less than 5	131 (46.6)
5-10	75 (26.7)
11-15	34 (12.1)
16-20	21 (7.5)
More than 20	20 (7.1)
**Clinical designation of the participants**	
Consultant	80 (28.5)
Specialist	16 (5.7)
Fellow	9 (3.2)
Resident/trainee/resident medical officer	119 (42.3)
Instructor/registrar/senior registrar	25 (8.9)
Medical officer/senior medical officer	32 (11.4)
**Administrative setup of the participant’s parent hospital**	
Public sector teaching hospital	95 (33.80)
Public sector non-teaching hospital	12 (4.3)
Private sector teaching hospital	160 (56.9)
Private sector non-teaching hospital	14 (5)

**Table 2.** Personal Protective Equipment in Common Practice

Personal Protective Equipment	Respondents n (%)
**Respiratory protection**	
Surgical mask	141 (50.2)
N95 Mask	214 (76.2)
Powered air-purifying respirator	91 (32.4)
Reusable respirator mask	123 (43.8)
**Contact precaution**	
Headcover/cap	192 (68.3)
Full medical protective suit	189 (67.3)
Long sleeves waterproof gown	198 (70.5)
Double gloving	264 (94)
Shoe covers	237 (84.3)
**Eye protection**	
Visor face shield	230 (81.9)
Goggles	181 (64.4)

**Table 3.** Important Guideline Statements and Reported Compliance

Guidelines	Respondents Compliance (%)
Limit staff presence to 3; 1 intubator, 1 assistant, and 1 to monitor patient and administer drugs	51.2
Use a tracheal intubation checklist.	37.4
Intubate in a negative pressure room with >12 air changes per hour when possible.	12.5
Best skilled airway manager should manage airway to maximize first pass success	54.1
Videolaryngoscopy for tracheal intubation	24.2
Two-handed mask ventilation with a V-E grip to improve seal	22.8
Second-generation supraglottic airway device for airway rescue	38.1
Do not mask ventilate unless needed	28.1
Confirm tracheal intubation with continuous waveform capnography	46.3
